# Host choice and human blood index of *Anopheles pseudopunctipennis *in a village of the Andean valleys of Bolivia

**DOI:** 10.1186/1475-2875-6-8

**Published:** 2007-01-22

**Authors:** Frédéric Lardeux, Paola Loayza, Bernard Bouchité, Tamara Chavez

**Affiliations:** 1 IRD, Caractérisation et Contrôle des Populations de Vecteurs, La Paz, Bolivia; 2 Laboratorio de Entomología Medica, Instituto Nacional de Laboratorios de Salud (INLASA), Casilla M-11, Miraflores, La Paz, Bolivia

## Abstract

**Background:**

The Human Blood Index (HBI, proportion of bloodmeals of a mosquito population obtained from man) is relevant to epidemiological assessment and to the modification of measures to interrupt malaria transmission since the vectorial capacity of the vector varies as the square of the HBI. *Anopheles pseudopunctipennis *is a main malaria vector in South America. Unfortunately, few data exist concerning HBI values in its range of distribution and none from Bolivia where this species is considered as an important malaria vector in the central Andes.

**Methods:**

The host choice of *An. pseudopunctipennis *has been studied in Mataral, a characteristic village of the central Andes of Bolivia. Mosquito host feeding preference experiments (equal accessibility to host in homogenous environment) were monitored using baited mosquito nets in latin square designs. Host feeding selection experiments (natural feeding pattern in heterogeneous environment) was measured by bloodmeal analysis, using ELISA to determine the origin of blood. Mosquito bloodmeals were collected on various occasions, using various techniques in a variety of sampling sites. A survey of the possible blood sources has also been carried out in the village. Data were analysed with the forage ratio method.

**Results:**

*An. pseudopunctipennis *chooses amongst hosts. Sheep, goats, donkeys and humans are the preferred hosts, while dogs, pigs and chicken are rarely bitten. *An. pseudopunctipennis *has an opportunistic behaviour, in particular within the preferred hosts. The HBI in Mataral is ≈40% and in the central Andes, may range from 30–50%, in accordance to other findings. A high proportion of mixed meals were encountered (8%), and cryptic meals are likely more numerous. There was no difference amongst the HBI from parous and nulliparous mosquitoes.

**Conclusion:**

Forage ratio analysis is a powerful tool to interpret mosquito host choices. However, refinements in sampling strategies are still needed to derive accurate and precise HBIs that could be computed to compare or follow epidemiological situations. The low antropophily of *An. pseudopunctipennis*, associated with changing environmental conditions, leads to unstable malaria (*Plasmodium vivax*) transmission in the central Andes. The opportunistic behaviour of this vector may be used to attract mosquitoes to insecticide. Zooprophylaxis is a promising alternative control strategy.

## Background

*Anopheles pseudopunctipennis *is a major vector of malaria in the Americas. Its geographical distribution extends from the United States (south of 40°N), Mexico, through Central America and the Andean countries of South America down to the northern part of Argentina (30°S), with an eastern extension into Venezuela and the Lesser Antilles. It is present in the foothills and mountainous areas of these countries, and above 600 m, it is often the only malaria vector. In Bolivia, it is present in the valleys of the Andean piedmont, up to an altitude of 2,800 m and transmits *Plasmodium vivax*. Despite numerous studies on its taxonomic, genetic and vector status, as well as insecticide effects on control strategies since the appearance of DDT, very few data are available on *An. pseudopunctipennis *ecology, and in particular on its host preference and host selection for blood feeding. Neither the Human Blood Index (HBI)(the proportion of blood meals taken on man), nor *a*, the proportion of blood meal taken on human per mosquito per 24 h (*a *= *HBI*/*duration of the gonotrophic cycle*) have ever been studied in details for *An. pseudopunctipennis*, despite the major importance of these factors in the understanding of malaria transmission. These parameters appear in the computation of entomological indexes such as, for example, the vectorial capacity (*C*), which expresses the potential for malaria transmission. The vectorial capacity (or at least some of its components) is an important parameter to estimate when malaria transmission is to be understood and controlled [1,2]. It was defined by Garret-Jones [3] from formulas developed by Macdonald [4] and may be defined as the daily rate at which future inoculations arise from a currently infective case. One of the possible equations used for measuring it is:

*C *= *m*.*a*2·*p*^*x*^/(-*Log*(*p*))     (1)

Where

*a *= Proportion of bloodmeals taken on man by a mosquito per 24 hours

*m.a *= Number of bites per man per 24 hours (*m *= mosquito density)

*p *= daily survival rate of the mosquito

*x *= duration of the extrinsic cycle of the transmitted *Plasmodium sp*.

Because a mosquito has to take two bites on man to transmit malaria, (*C*) is sensitive to changes in the man biting rate (*ma*), and to *a*, the proportion of blood meals taken on man in 24 h, which appears as *m.a*^2 ^in the formula. Because the vectorial capacity varies as the square of the frequency of feeding (*a*^2 ^in the *C *formula), and likewise as the square of the HBI, small changes in the HBI may have a significant impact on operational control of transmission. The *a *parameter may also be useful to compute the malaria stability index [4]:-*a*/*Log(p)*. This basic analysis of the Ross-MacDonald model of malaria transmission reveals that along with the probability that a mosquito will survive long enough to transmit, the probability of biting on human is the second most important parameter.

The estimation of *a *(or the HBI) alone is always troublesome [5]. However, blood meal identifications provide an integral part of epidemiological studies. The prevalence of malaria in an area is greatly influenced by the process of host selection by malaria vectors which, in turn, is influenced by many factors including host preferences of the vector [6]. A good simple example of the influence of *a *on potential for malaria transmission is given by the comparison of *Anopheles arabiensis *with *Anopheles gambiae *in Africa. *An. gambiae *species complex is one of the most highly anthropophilic, while *An. arabiensis *is an opportunistic species that feeds preferentially on humans in many parts of Africa, but can be diverted to domestic animals as their density increases. As such, in certain areas, this less antropophilic pattern leads to a low mean annual sporozoite rate of about 1–2% compared to *An. gambiae *which shows a mean annual sporozoite rate of 6% [7-9].

For *An. pseudopunctipennis*, very few data exist on its host feeding patterns, and in general terms, studies lack rigorous sampling and data analysis. In Argentina, the HBI was found to be 50% [10], in Venezuela, a small sample gave 2.2% [11]. Indoor mosquitoes from Mexico gave an HBI of 67% [12] and in Peru, the HBI ranged from 40–50% to 85% [13,14]. Unfortunately, all these estimates lacked data from the surrounding available host densities and as such, only point out the variability of the HBI within sampling areas. Others found that *An. pseudopunctipennis *was indeed anthropophilic but as well was able to feed on animals [15]. *An. pseudopunctipennis *from Mexico, El Salvador and Costa Rica took bloodmeals from bovids and equiids (39%-22%; 60%-12%; 78%-0% respectively), but because of inadequate sampling, data could not be used to derive any HBI [16]. In villages of Mexico, 54% to 86% of engorged females resting inside houses were human-blood fed; in all captured resting females (inside and outside houses) humans and dogs were the more common blood sources and the HBI ranged from 29 – 55% [17]. Comparing trapping methods, a horse-baited trap was more effective than human landing catches [18]. Another study carried out in Mexico showed that the HBI ranged from 3.3 – 6.8% in DDT sprayed houses, because of irritability and repellence of the chemical which limited mosquito entrance in the houses [19]. These few data only demonstrate the ability of *An. pseudopunctipennis *to feed on a variety of blood sources, and to change its targets when ecological factors change. *An. pseudopunctipennis *live in various ecological situations, with different habits according to regions [20]. Because of the variability of environmental confounding factors that influence feeding patterns, it is often impossible to compute a general estimate of feeding preference to be used in the whole distribution range of the species. As such, the present study only claims to evaluate the feeding patterns of *An. pseudopunctipennis *in a characteristic situation in the central Andes of Bolivia, and interpret the results in terms of malaria transmission risks in that area where similar conditions occur.

## Methods

### Study area

Experiments where carried out in Mataral (S 18.6024, W 65.1444) at an altitude of approximately 1,500 m. Mataral is a small village characteristic of those encountered in the dry valleys of the Bolivian Andes, where *An. pseudopunctipennis *transmits *P. vivax*. It is situated in the centre of Bolivia, at about 100 km north of the constitutional capital Sucre. There, about 1,000 inhabitants live in about 80 grouped houses. They are subsistence small farmers and most of their animals (mainly goats and sheep) are kept nightly in small corrals close to the houses. Pigs, some cows and donkeys wander in the village, as well as dogs and chickens. The village is close to a small river which is used as a water source for the inhabitants and their cattle, and where *An. pseudopunctipennis *larval habitats are found. The climate is xeric, characterized by a mean annual temperature of 18°C, with daily maximum of 39°C during the austral summer and 5°C during winter (July – August) [21]. The monthly mean temperatures are above 15°C. Rainfalls are short and violent, and occur mainly between November and March. Their annual mean is between 400 and 600 mm.

### Host choice experiments

Blood feeding of mosquitoes depends roughly on two components: host preference and host selection [22]. Host preference is defined as the choice of a particular vertebrate host as a food source, rather than other species equally available. It is an intrinsic physiological characteristic of the insect that does not take into account host availability or host irritability. On the contrary, host selection is related to a pattern of feeding in nature, as shown by the relative frequency of blood of different types from a mosquito population in time and space, and may be influenced by ecological factors.

In our study, these two components were considered. To measure host preference, hosts should be equally accessible, with environment and ecological conditions as homogenous as possible at the time of the experiment. This could be achieved by presenting possible hosts closed to each other (in mosquito-nets, for example) and analyzing the number of mosquitoes choosing one or another bait.

When host selection is to be analysed, a real field situation should be experienced. In the field, not all the possible hosts are present at the same time in the same place. So, mosquitoes may choose a "second choice" host because the preferred host is not present or not accessible. Accessibility to hosts may be biased by ecological conditions (meteorological factors, host abundance, accessibility, dispersion and geographical repartition etc.). Host selection is then dependent on host preference, but also on the probability of host encounter, with *a priori*, a positive relationship between host preference and host selection: the more intense is the preference for one host type and the more frequent will be the blood feedings on that host, because mosquitoes will search specifically for that one. If the mosquito species is not highly specialized in one host type (i.e., is "opportunistic" in its host choice), it will not search for a preferred host when another blood source is available. Host selection experiments may analyse this situation and separate factors of host selection into its components of host preference and random selection.

### Host feeding preference experiments (equal accessibility to host in homogenous environment)

Host preference experiments were carried out on various occasions to estimate the feeding preferences of *An. pseudopunctipennis *when accessibility to hosts is equal. In each experiment, animal baits were disposed outdoors under mosquito net traps and mosquitoes were regularly sampled during the night. Mosquito nets were disposed close to *An. pseudopunctipennis *larval sites, side by side, at some meters intervals, on the river banks which offered space and no evident "other hosts" attraction for mosquitoes. Each experiment was latin-square designed. The Latin square design is used where the researcher desires to control the variation in an experiment that is related to rows and columns in the field. Treatments (baits) are assigned at random within rows (nights) and columns (mosquito net locations), with each treatment once per row and once per column. For example, with four hosts, the experiment needed four mosquito nets and lasted four nights. Baits were chosen amongst: man (under double mosquito net to avoid mosquito bites), donkey, goat, sheep, pig, cow, dog and chicken (Table 1). Donkeys, cows and pigs were chosen as young small animals. Experiments were carried out with one cow, one donkey or one pig, two goats, two sheep or two dogs, and five chickens at a time under mosquito nets.

**Table 1:** Host feeding preference experiments with baited mosquito-nets

Date	April 2002	May 2002	June 2002	July 2002	August 2002	August 2002	April 2003	May 2003
Number of nights sampled	3	1	3	4	3	1	1	4

Man	24	17	53	22	36	13	21	322
Cow	49							
Donkey	114	37		42			8	162
Sheep			89	56			13	90
Goat		13	26	20				52
Pig					15	5		
Dog						1		
Chicken					0	0		
Empty net		1						
% of mosquitoes attracted to "man"	12.8	25	31.5	15.7	70.6	68.5	50	51.4
% of mosquitoes attracted to "man"(weighted)	18	24.7	18.7	10.9	54.5	52	40.1	45.1

Total number of mosquitoes captured under baited mosquito-nets during various studies in Mataral. The percentages of mosquitoes attracted to "man" are calculated with rough and weighted data (see text).

### Host feeding selection experiments (natural feeding pattern in heterogeneous environment)

Engorged mosquitoes where collected at various sites in the village, during various occasions and using various methods such as (Table 2): Mosquito resting collections (from natural and artificial shelters), volunteer human bait collections and CDC light traps. Blood engorged resting mosquitoes were captured in 200 litre-drums disposed horizontally at 50 cm above ground level and covered with soil to regulate temperature inside. Two of these shelters were installed outdoors at two different locations in the village. Another resting shelter (a natural cave) was situated at one end of the village. It consisted in a small cave of about 2 × 2 × 2 m in a clay cliff. There, mosquitoes were collected during the night until dawn, in small hollows dug in the cave's walls.

**Table 2:** Origin of blood-fed mosquitoes captured in Mataral

	Collection method							
	
Host	CDC light trap	Outside Houses (human landings)	Inside House (human landings)	Residual fauna	Cave	Drum-trap 1	Drum-trap 2	TOTAL
Donkey (Horse)	0	2 (1)	0	1	27 (2)	1	5 (2)	36 (5)
Dog	1	4 (1)	4 (1)	0	10 (2)	1	2 (2)	22 (6)
Human	1	30 (2)	25 (8)	9	31 (3)	2	7 (3)	105 (16)
Goat (Sheep)	4	19 (4)	22 (7)	5	88 (13)	5	36 (3)	179 (27)
Pig	0	3 (1)	4 (1)	0	3 (1)	1	7	18 (3)
Chicken	0	0 (2)	0	0	0	0	1	1 (2)
Cow	1	1 (1)	3 (1)	0	5 (5)	0	2	12 (7)
Other (unidentified)	1	0	0	0	7	2	1	11
Total	8	59 (12)	58 (18)	15	171 (26)	12	61 (10)	384 (66)
HBI		45.1%	43.4%	60.0%	17.2%	16.7%	14.1%	26.8%

Number of single bloodmeals and mixed bloodmeals (in brackets) per host and collection method. The total number of bloodmeals per category should be calculated as number of single meals + number of mixed meals/2

Volunteer human bait collections were part of another study and mosquitoes were also analysed at this occasion for blood meal origin, taking into account the following: Four different houses of the village were sampled (indoor and outdoor) each night (18h00 – 6h00) during four consecutive nights on a monthly basis. Each night, the houses were changed. Each hour of the night, collected mosquitoes were identified and dissected for parity and for Chistopher's ovary stage classification. Blood encountered in those mosquitoes was processed. The result was carefully analysed when blood was fresh (bright red colour of the blood) in a fully engorged mosquito, indicating that the captured mosquito may have bitten the volunteers. If doubtful the blood meal was discarded. If digested (*i.e. *blood in lower quantity, brownish colour), or if ovaries were at a development stage posterior at "II-late" in the Christopher's classification, the blood indicated that the meal was taken at least one day before. Some engorged mosquitoes were also captured with CDC light traps disposed in the village, and during some collections of morning resting fauna inside houses.

### Identification of bloodmeals

Each engorged mosquito captured was identified and classified according to its capture location and type (type of shelter, volunteer bait collection (inside or outside houses) etc.), level of engorgement and when possible, its Christopher's stage status. In the field, each blood meal was squashed on a filter paper and kept refrigerated (4°C) until laboratory processing. Blood meals were identified by the enzyme-linked-immunosorbent assay (ELISA) technique [23] modified as follows. Each blood smear was macerated in 800 μl 0.01 M Phosphate Buffer Saline (PBS) at pH 7.2 for one hour in 1 ml Eppendorf tube. 96-well ELISA plates (NUNC Maxisorp, flat bottom) were coated with the mosquito blood (50 μl/well; one column for one mosquito) and incubated three hours at room temperature. Positive controls were animal serums (at 1/100 in PBS) in column 12, and negative controls were PBS in column 1. The plate was then emptied and saturated with 200 μl/well of blocking buffer solution (casein: 5 g; NaOH [0.1 N]: 100 ml; PBS: 900 ml; Tween 20: 250 μl; thimerosal: 0.1 g; phenol red: 0.02 g) and incubated one hour at room temperature. The plate was emptied, washed twice with PBS/Tween 20 (0.05%) and 50 μl/well of peroxidase conjugated antibodies anti-IgG of each tested animal were added. The antibodies were prepared as follows: for each type of blood tested, 2 μl of peroxidase specific anti-IgG at 1/1,000 (human, cow, pig, dog, horse), 1/500 (goat) or 1/2,000 (chicken) were added to 2 ml of blocking buffer in 10 ml glass tubes (one tube/blood type) and mixed with 2 μl of each corresponding heterologous serums to avoid cross-reactions. The plate was incubated for one hour at room temperature, emptied and washed four times with PBS/Tween. Then 100 μl of peroxidase substrate (orthotolidin: 0.01 g; N-N-dimethyl-formamid: 250 μl, citrate buffer pH 4 (citric acid 11.77 g, sodium hydroxide 4.48 g, distilled water 1 l): 60 ml, H_2_O_2_: 120 μl) was added in each well and the plate was incubated 30 min in the dark at room temperature. 50 μl/well of sulphuric acid 4N were added to stop the reaction and absorbance values were obtained at 450 nm in a ELISA reader (Multiscan). In general terms, positive wells (those in which blood meal antigen and antispecies antibody homologous react) showed a yellowish colour more pronounced than did the negative control wells. Samples were considered positive if absorbance values exceeded the mean value + 2 standard deviations of the 10 negative controls. All chemicals came from Sigma-Aldrich, Lyon, France. As there are cross-reactions between horse and donkey on one hand and sheep and goat on the other hand, these animals were not separated by species.

### Statistical analysis

#### Host feeding preference when accessibility to hosts is equal

The latin-square designs with mosquito-nets for studying host selection were analysed with ANOVA. Probabilities of the F tests were at *α *= 0.05 level. Field data were transformed according to host weight [24]. Due to animal size and the number of animals used under each mosquito-net in our experiments, weighting coefficients were 1 for "man", 0.67 for "cow" and "donkey", 2 for "dog", "goat", "pig", "sheep" (two of these animals were used as one single bait) and 20 for "chicken" (five chicken were used as one bait). Prior to analysis, data were transformed to Log(x+1) to homogenize and stabilize the variances. Forage ratios (see below) were also used to indicate which baits were more attractive in each experiment.

#### Host feeding selection in heterogeneous environment

In this case, the problem is to estimate the probabilities of selection of each host type or a set of preferences that are proportional to these probabilities that are often difficult to calculate in an absolute way. As such, it is possible to conclude for example that *An. pseudopunctipennis *prefers to feed on "that type" of animal, without knowing exactly what fraction of these animals are in fact bitten by the mosquitoes in the area. A simple and efficient method to measure preference of mosquitoes for blood resources, is the computation of the forage ratio index [25], named also "Index2" of Cock [26] or "selection index" of Manly *et al. *[27]. The forage ratio *w*_*i *_for species *i *is:

wi=oipi     (2)
 MathType@MTEF@5@5@+=feaafiart1ev1aaatCvAUfKttLearuWrP9MDH5MBPbIqV92AaeXatLxBI9gBaebbnrfifHhDYfgasaacH8akY=wiFfYdH8Gipec8Eeeu0xXdbba9frFj0=OqFfea0dXdd9vqai=hGuQ8kuc9pgc9s8qqaq=dirpe0xb9q8qiLsFr0=vr0=vr0dc8meaabaqaciaacaGaaeqabaqabeGadaaakeaacqWG3bWDdaWgaaWcbaGaemyAaKgabeaakiabg2da9maalaaabaGaem4Ba82aaSbaaSqaaiabdMgaPbqabaaakeaacqWGWbaCdaWgaaWcbaGaemyAaKgabeaaaaGccaWLjaGaaCzcamaabmaabaGaeGOmaidacaGLOaGaayzkaaaaaa@3A7B@

Where

*w*_*i *_= forage ratio for species *i*

*o*_*i *_= proportion or percentage of species *i *in the blood meals

*p*_*i *_= proportion or percentage of species *i *available in the environment

In the case of mosquitoes resource preference, "species *i*" refers to blood sources, i.e., humans or animals such as cows, dogs, pigs etc.

Index values above 1 indicate preference and less than 1 indicate avoidance. As indices may range from 0 to infinity, which is a nuisance, Manly *et al. *[27] suggested presenting standardized forage ratios (*B*_*i*_) so that their sum equal 1 for all resource types

Bi=wi∑i=1nwi     (3)
 MathType@MTEF@5@5@+=feaafiart1ev1aaatCvAUfKttLearuWrP9MDH5MBPbIqV92AaeXatLxBI9gBaebbnrfifHhDYfgasaacH8akY=wiFfYdH8Gipec8Eeeu0xXdbba9frFj0=OqFfea0dXdd9vqai=hGuQ8kuc9pgc9s8qqaq=dirpe0xb9q8qiLsFr0=vr0=vr0dc8meaabaqaciaacaGaaeqabaqabeGadaaakeaacqWGcbGqdaWgaaWcbaGaemyAaKgabeaakiabg2da9maalaaabaGaem4DaC3aaSbaaSqaaiabdMgaPbqabaaakeaadaaeWbqaaiabdEha3naaBaaaleaacqWGPbqAaeqaaaqaaiabdMgaPjabg2da9iabigdaXaqaaiabd6gaUbqdcqGHris5aaaakiaaxMaacaWLjaWaaeWaaeaacqaIZaWmaiaawIcacaGLPaaaaaa@411E@

Where

*B*_*i *_is the standardized selection index for species *i*

*w*_*i *_= forage ratio for species *i *(from eq. 2)

*n *= number of types of blood, i.e., number of different blood sources available

As such, standardized forage ratio values of (1/*n*) indicates no preference. Values below this indicate relative avoidance, and values above indicate relative preference.

A complete census of available blood resources for mosquitoes has been carried out in Mataral (Table 1), and as such, to test the null hypothesis that mosquitoes are selecting resources at random, the following G-test [27] was used, computing the following χ^2 ^value with (*n*-1) degree of freedom (H_0 _= random selection)

χ2=2∑i=1n[ui.ln⁡(uiU.pi)]     (4)
 MathType@MTEF@5@5@+=feaafiart1ev1aaatCvAUfKttLearuWrP9MDH5MBPbIqV92AaeXatLxBI9gBaebbnrfifHhDYfgasaacH8akY=wiFfYdH8Gipec8Eeeu0xXdbba9frFj0=OqFfea0dXdd9vqai=hGuQ8kuc9pgc9s8qqaq=dirpe0xb9q8qiLsFr0=vr0=vr0dc8meaabaqaciaacaGaaeqabaqabeGadaaakeaacqaHhpWydaahaaWcbeqaaiabikdaYaaakiabg2da9iabikdaYmaaqahabaWaamWaaeaacqWG1bqDdaWgaaWcbaGaemyAaKgabeaakiabc6caUiGbcYgaSjabc6gaUjabcIcaOmaalaaabaGaemyDau3aaSbaaSqaaiabdMgaPbqabaaakeaacqWGvbqvcqGGUaGlcqWGWbaCdaWgaaWcbaGaemyAaKgabeaaaaGccqGGPaqkaiaawUfacaGLDbaaaSqaaiabdMgaPjabg2da9iabigdaXaqaaiabd6gaUbqdcqGHris5aOGaaCzcaiaaxMaadaqadaqaaiabisda0aGaayjkaiaawMcaaaaa@4EC0@

Where

*u*_*i *_= Number of mosquitoes with blood of type *i*

U=∑i=1nui
 MathType@MTEF@5@5@+=feaafiart1ev1aaatCvAUfKttLearuWrP9MDH5MBPbIqV92AaeXatLxBI9gBaebbnrfifHhDYfgasaacH8akY=wiFfYdH8Gipec8Eeeu0xXdbba9frFj0=OqFfea0dXdd9vqai=hGuQ8kuc9pgc9s8qqaq=dirpe0xb9q8qiLsFr0=vr0=vr0dc8meaabaqaciaacaGaaeqabaqabeGadaaakeaacqWGvbqvcqGH9aqpdaaeWbqaaiabdwha1naaBaaaleaacqWGPbqAaeqaaaqaaiabdMgaPjabg2da9iabigdaXaqaaiabd6gaUbqdcqGHris5aaaa@38CC@ = total number of blood meal types in all the mosquitoes blood meals

*n *= number of blood resources categories

To give statistical significance to the forage ratios, the standard error *s*_*wi *_of *w*_*i *_were computed as:

swi=oi.(1−oi)U.Pi2     (5)
 MathType@MTEF@5@5@+=feaafiart1ev1aaatCvAUfKttLearuWrP9MDH5MBPbIqV92AaeXatLxBI9gBaebbnrfifHhDYfgasaacH8akY=wiFfYdH8Gipec8Eeeu0xXdbba9frFj0=OqFfea0dXdd9vqai=hGuQ8kuc9pgc9s8qqaq=dirpe0xb9q8qiLsFr0=vr0=vr0dc8meaabaqaciaacaGaaeqabaqabeGadaaakeaacqWGZbWCdaWgaaWcbaGaem4DaCNaemyAaKgabeaakiabg2da9maakaaabaWaaSaaaeaacqWGVbWBdaWgaaWcbaGaemyAaKgabeaakiabc6caUiabcIcaOiabigdaXiabgkHiTiabd+gaVnaaBaaaleaacqWGPbqAaeqaaOGaeiykaKcabaGaemyvauLaeiOla4Iaemiuaa1aa0baaSqaaiabdMgaPbqaaiabikdaYaaaaaaabeaakiaaxMaacaWLjaWaaeWaaeaacqaI1aqnaiaawIcacaGLPaaaaaa@4635@

And the confidence limits were calculated in the usual way as:

*w*_*i *_± *Z*_*α*_·*S*_*wi *_    (6)

Where *z*_*α *_is the standard normal deviate. The Bonferonni correction [28], which corrects for multiple comparisons, was applied to maintain a consistent overall error rate (*α*/*n *is used instead of *α *for computing *z*).

Forage ratios were computed in three ways, using values of *oi *and *pi *from direct counts of animals, or taking into account the weight of animals [24], or using the transformed data Log(*pi*+1) to take into account that animals are not dispersed at random on the whole range of influence of *An. pseudopunctipennis *in the village. For example, 1,002 goats and sheep were counted in the village (Table 3). However, these animals did not wander during the nights. They are kept in groups of 20–80 individuals in small corals close to houses. Dogs, pigs, chicken etc. also sleep in groups. Humans usually slept as one family grouped in one room. The Log transformation tends to diminish the weight of large values in the computations (such as the high number of goats), and as such acts as if the number of individuals hosts was reduced due to grouping.

**Table 3:** Bloodmeal sources of *An. pseudopunctipennis *in Mataral

				Computation with rough data on host abundance		Computation with Log transformed data on host abundance	
	Bloodmeals	Bloodmeals including mixed meals	Host abundance	Forage ratio (95%confidence interval)	Standardized forage ratio of Manly et al. (1993)	Forage ratio (95%confidence interval)	Standardized forage ratio of Manly et al. (1993)

Single host					Threshold: 0.143		Threshold: 0.143
Human	105	121	228	2.00 (1.70 – 2.31)	0.030	1.47 (1.25 – 1.69)	0.200 *
Goat or Sheep	182	210	1002	0.79 (0.71 – 0.87)	0.012	1.99 (1.80 – 2.19)	0.271 *
Donkey or Horse	36	41	4	38.74 (27.4 – 50.0)	0.571 *	1.95 (1.38 – 2.52)	0.265 *
Dog	22	28	101	1.05 (0.67 – 1.42)	0.015	0.40 (0.26 – 0.54)	0.054
Pig	19	22	68	1.22 (0.73 – 1.72)	0.018	0.34 (0.20 – 0.48)	0.047
Cow	11	19	3	23.93 (13.40 – 34.46)	0.353 *	1.15 (0.65 – 1.66)	0.157 *
Chicken	1	3	272	0.04 (0 – 0.09)	0.001	0.03 (0 – 0.75)	0.005
Other (unidentified)	11	11					
							
Mixed blood meals							
Human/Goat	10						
Human/Donkey	1						
Human/Dog	2						
Human/Pig	1						
Human/Chicken	2						
Goat/Donkey	4						
Goat/Dog	4						
Goat/Pig	2						
Goat/Cow	8						

Number of single and patent mixed blood meals analysed in Mataral, number of domestic hosts present and forage ratios with rough and Log-transformed host numbers (95% confidence intervals in brackets). * indicate the preferred host.

When percentages were computed, they were compared using the χ^2 ^statistic at the *α *= 0.05 significance level.

## Results

### Host preference in homogenous environment

Eight experiments were carried out with baited mosquito-nets of which five were during successive nights and thus enabled ANOVA analysis (Table 1). The first experiment (April 2002) compared host preferences amongst "man", "donkey" and "cow" during three consecutive nights. There was no difference between the number of *An. pseudopunctipennis *captured each night (*F *= 4.32; df = 2; *P *= 0.18) nor between the mosquito net locations (*F *= 0.21; df = 2; *P *= 0.82), but a slight difference between the baits (*F *= 19.21; df = 2; *P *= 0.049). The "man" was less attractive with a weighted mean of 8 mosquitoes, while the "donkey" attracted a weighted mean of 25.5 mosquitoes and the "cow" 10.9. Forage ratios indicated that *An. pseudopunctipennis *made a choice and that the donkey was the most attractive (Table 4).

**Table 4:** Forage-ratios for each baited mosquito-net experiment

	April 2002	May 2002	June 2002	July 2002	August 2002	August 2002	April 2003	May 2003
Man	0.30 (0.19 – 0.41)	0.93 (0.55 – 1.31)	1.57* (1.22 – 1.92)	0.89 (0.55 – 1.23)	2.12* (1.74 – 2.49)	3.42* (2.37 – 4.46)	1.83* (1.28 – 2.39)	2.92* (2.70 – 3.14)
Cow	0.91 (0.70 – 1.13)							
Donkey	2.13* (1.88 – 2.37)	3.02* (2.37 – 3.67)		2.54* (1.89 – 3.18)			1.04 (0.39 – 1.69)	2.19* (1.90 – 2.48)
Sheep			1.32* (1.13 – 1.51)	1.13 (0.90 – 1.36)			0.57 (0.31 – 0.82)	0.41 (0.33 – 0.48)
Goat		0.35 (0.18 – 0.53)	0.39 (0.25 – 0.52)	0.40 (0.24 – 0.57)				0.23 (0.17 – 0.30)
Pig					0.44 (0.25 – 0.63)	0.66 (0.16 – 1.15)		
Dog						0.13 (0 – 0.38)		
G-test	105.6 (df = 2, *P *= 0)	52.6 (df = 2, *P *= 0)	48.9 (df = 2, *P *= 0)	51.1 (df = 3, *P *= 0)	29.4 (df = 1, *P *= 0)	23.7 (df = 2, *P *= 0)	11.47 (df = 2, *P *= 0.)	631.4 (df = 3, *P *= 0)

Forage ratios and 95% confidence intervals (in brackets) for each monthly mosquito-net experiment. Forage ratios were computed using host weight weighting. Values above 1 indicate preference and <1 avoidance. * indicate values statistically above 1. *P *< 0.05 with the G-test indicate that *An. pseudopunctipenni*s made a choice amongst the proposed hosts during the experiment.

A second experiment carried out in June 2002 compared host preferences amongst "man", "sheep" and "goat" during three consecutive nights. There was no difference amongst the "nights" (*F *= 1.18; df = 2; *P *= 0.35) nor the "mosquito net locations" (*F *= 0.80; df = 2; *P *= 0.55), but again amongst the baits (*F *= 20.6; df = 2; *P *= 0.046). The "sheep" was the most attractive with a weighted mean of 59.3 mosquitoes, while the "man" and "goat" attracted weighted means of 17.7 and 17.3 mosquitoes respectively. Once again, the forage ratio G-test indicated that the mosquito chose its hosts and in that experiment, the "man" and "Sheep" were the preferred host (Table 4).

A third experiment carried out in July 2002 compared "man", "donkey", "sheep" and "goat" in a 4-night design. There was no difference amongst the "nights" (*F *= 2.57; df = 3; *P *= 0.14) nor the "mosquito net locations" (*F *= 0.47; df = 3; *P *= 0.71), but again amongst the baits (*F *= 5.80; df = 3; *P *= 0.03). The "sheep" was the most attractive with a weighted mean of 28.0 mosquitoes captured. The "goat", "donkey" and "man" attracted weighted means of 10.0, 7.0 and 5.5 mosquitoes respectively. The forage ratio G-test indicated that *An. pseudopunctipennis *chose preferably amongst its hosts (Table 4).

A fourth experiment carried out in August 2002 compared the attractiveness of "man", "pig" and "chicken". Once again, there was no differences amongst the "nights" (*F *= 1.52; df = 2; *P *= 0.39) nor the "mosquito-net locations" (*F *= 0.09; df = 2; *P *= 0.92), but again amongst the baits (*F *= 20.9; df = 2; *P *= 0.045). The "chicken" was the less attractive (0 mosquitoes captured), while the "man" and "pig" attracted a weighted mean of 12.0 and 10.0 mosquitoes. Forage ratios indicated that the "man" was the preferred host (Table 4).

A fifth experiment in May 2003 compared host preference amongst "man", "donkey", "sheep" and "goat". There were no difference amongst the "night" (*F *= 0.34; df = 3; *P *= 0.79) nor the "mosquito-net locations" (*F *= 1.77; df = 3; *P *= 0.25), but again amongst the baits (*F *= 4.84; df = 3; *P *= 0.048). Unlike July 2002 when the same baits where proposed, the "man" was here the most attractive with a weighted mean of 80.5 mosquitoes captured, while the "sheep", "donkey" and "goat" attracted a weighted mean of 45.0, 27.2, and 26.0 mosquitoes respectively. Forage ratios values and the G-test indicated that the mosquito preferred "man" and "donkey" to the other proposed baits (Table 4).

Due to weather conditions on three occasions (May 2002, August 2002 and April 2003), experiments lasted only one night (Tab. 1). Although it was not possible to analyse by ANOVA these results, the forage ratios and G-tests indicated that *An. pseudopunctipennis *chose amongst hosts. "man" was selected when opposed to "pig", "dog", "sheep" or "goat". The "donkey" was also a selected host (Tab.4).

*An. pseudopunctipennis *host choice is more or less pronounced, depending on the panel of hosts proposed and possibly on the season: The weighted proportions of mosquitoes feeding on "man" when compared to animals ranged from 10.9% to 54.5% (Table 1).

### Host preference in heterogeneous environment

The overall HBI calculated from bloodmeal data including mixed meals was 26.6% and 27.1% when computed with only the single meals (Tab. 3). Depending on the collection method, the HBI ranged from 14.1% (in Drum-traps) up to 60.0% (Morning indoors resting collections). Overall forage ratios computed with rough and Log-transformed data are presented in Table 3. The G-tests computed with rough and Log-transformed data were 520 (df = 6; *P *= 0) and 336 (df = 6; *P *= 0) respectively, indicating that *An. pseudopunctipennis *did made a choice amongst the possible hosts. Preferred hosts were "donkeys -horses", "goats -sheep", "humans" and "cows" in that order.

The proportion of blood meals taken on humans by parous and nulliparous *An. pseudopunctipennis *females were 32.3% and 23.4% respectively. These two percentages did not differ significantly (χ^2 ^= 1.22, df = 1, *P *= 0.27). The proportion of parous *An. pseudopunctipennis *that had taken a bloodmeal on a human and come back to a human was 77.3%, and was not significantly different from the proportion of parous females with bloodmeals from humans (71.4%) found from other sampling sources (cave, 200 l-drums, CDC light traps) (χ^2 ^= 0.19, df = 1, *P *= 0.66) (Table 5).

**Table 5:** Bloodmeal sources from parous and nulliparous *An. pseudopunctipennis *in Mataral

	*An. pseudopunctipennis *from human landing catches		*An. pseudopunctipennis *from other sources of sampling	
	
	N° of bloodmeals taken on humans (percentage)	N° of bloodmeals taken on animals (percentage)	N° of bloodmeals taken on humans (percentage)	N° of bloodmeals taken on animals (percentage)
From parous females	17 (77.3%)	19 (73.1%)	15 (71.4%)	48 (63.2%)
From nulliparous females	5 (22.7%)	7 (26.9%)	6 (28.6%)	28 (36.8%)
Total	22	26	21	76

Number and percentage (in brackets) of bloodmeals from parous and nulliparous *An. pseudopunctipennis*, from human landing catches (i.e. from mosquitoes coming back to bite humans), and from other sources of sampling (cave, 200 l-drums, CDC light-traps)

There was a slight difference amongst HBIs computed by months (when sufficient data were available, i.e. 25 – 57 mosquitoes in January, May, July and October). There was a linear increase in the HBI values from January to October, computed from either mosquitoes captured in the cave or from human landings (HBI range 10.5 – 33.3% and 31.3 – 61.1% respectively; χ^2 ^= 8.39, df = 3, *P *= 0.04 and χ^2 ^= 9.13, df = 3, *P *= 0.03 respectively).

### Multiple bloodmeals

#### Multiple bloodmeals highlighted by blood analysis

Multiple bloodmeals result from two or more feeds, the last of which has been taken before the first one has been entirely digested. The ELISA technique enables us to identify only patent multiple feeds, i.e., mixed bloodmeals taken on two different hosts, but not cryptic mixed meals i.e., taken on the same host species. The ELISA analysis of bloodmeals indicated that 34 mosquitoes presented patent mixed blood meals (i.e., 8% of the bloodmeals) (Table 3). The contribution of hosts types to mixed meals reflects their distribution in single bloodmeals (χ^2 ^= 1.54, df = 4, *P *= 0.82), except for cows for which, patent mixed meals were over-represented (eight mixed meals and only 11 single meals).

### Multiple blood meals highlighted by bloodmeal analysis of human landing mosquitoes

The 132 mosquitoes collected by human landing catches and with bloodmeals remains may be considered as insects about to take a meal on a human. Fifteen (11.3%) mosquitoes already had a mixed blood (10 with human blood) when landing on humans. Taking into account these patent mixed meals, the *Anopheles *totalled 147 bloodmeals of which 65 were taken on humans and 82 (from 68 mosquitoes) were on animals. As such, the probability for a mosquito to take a second bloodmeal on human while a first one was also taken on human is 0.44 (65/147). The probability of taking a bloodmeal on a human while a first one was on an animals is then 0.56 (82/147). The two probabilities were not significantly different from 0.5 (χ^2 ^= 1.98, df = 1, *P *= 0.30). From the 132 mosquitoes captured by human landings, 98 were classified following Chistopher's ovary classification. Only digested blood was taken into account and, in mosquitoes with ovaries in stage II, only fresh meals that were not identified as "human", to avoid over-estimation of "human" blood from mosquitoes which might have begun to feed on the volunteers. As such, mosquitoes with ovaries in stage >III (40%) were considered as mosquitoes with blood taken from a preceding night, while others (ovaries in stage II) (60%) from immediate interrupted feeding. In former category, the proportion of human blood was 43;6% (56.4% animals), and in the later category, 49% (51% animals).

## Discussion

Host choices are genetically based, mosquitoes responding to particular hosts cues [29], but in nature, the expression of host preference depends also on the influence of many confounding factors. Characterizing mosquito host choice with the two parameters of host preference and host selection is useful but may be more complicated than that. Relevant parameters may be distinguished as follows. Determining parameters may be as follow. The probability for one type of host to be bitten can separated in two major components. The first one, named here "host availability", quantifies all the ecological, biological and behavioural factors that may modify the probability for one type of host to be bitten. The second one, called here "biting power" of the mosquito, groups all the intrinsic factors of the mosquito.

Availability can also be divided into two components. On one hand, the "accessibility", which is related to the host density in the area, and on the other hand, the "vulnerability", which depends on the interactions on hosts and mosquitoes and are often the consequence of their behaviour (vulnerability is, for example, correlated to the flight range of the mosquito). Practically, it may be difficult to distinguish between these two components but in general terms, they act at two different spatio-temporal scales. Variations in accessibility are usually on a season scale basis (i.e., migrations of hosts) while vulnerability factors vary at a smaller scale level (i.e., at the night level, inducing changes in host behaviour that may for example, move from the areas of high mosquito densities).

The other major component, the "biting power" of the mosquito, quantifies all the mosquito's intrinsic factors that make it more or less attracted to one type of host. These factors may have a genetic basis and are expressed through the physiology and behaviour of the mosquito. As such, when computing for example the HBI, results depend on (i) the number of humans present, (ii) the variations in their "availability" and (iii) the variations in the "biting power" of the mosquito species. The "biting power" for one type of host is composed of two different aspects. The first one is related to the mosquito host preference as defined by Boreham and Garrett-Jones [22]. The second one is the ability of the mosquito to bite a specific host, and covers two notions that should be distinguished: the biting efficiency and the biting effectiveness.

The biting efficiency corresponds to the ability for one mosquito to encounter vulnerable hosts and is roughly related to the notion of host selection, while the biting effectiveness can be defined as the ability to bite vulnerable hosts and is more depending on the behaviour of hosts and the ability of the mosquito to change to alternative hosts when disturbed.

Based on these new concepts, the "host selection" depends on both the "host availability" and "mosquito biting power". So, mechanisms underlying the computation of the HBI (or other similar index) are numerous and often difficult to quantify. The consequence of the numerous sources of variability is that host-vector contact is far from randomly distributed [6] and these interactions between genetic and environmental components make the estimation of feeding patterns difficult [30]. Moreover, mosquito sampling techniques and sampling locations may introduce biais in computations [31]. For example, fixed sampling places may capture biased subpopulations of mosquitoes [32]. Sampling resting mosquitoes indoors may over-estimate an HBI. On the contrary, sampling resting mosquitoes too close to a corral with animals may under-estimate an HBI if the mosquito species is not strongly anthropophagic. Because the HBI of a population can only be assessed by field sampling, it is hardly possible to know whether the samples are fully representative.

In Mataral's samples (indoors, outdoors, cave, 200 l-drums etc.), the HBIs ranged from 14–60%, and the true HBI would then depend on a proper combination of the values in the various biotopes. In Mataral, the cave was located at one end of the village and was more favourable to shelter resting mosquitoes which have fed on wandering cattle than on man. On the contrary, mosquitoes collected inside houses during morning resting catches may overestimate the HBI. In the Mataral experiments, the HBI's least biased value may be that calculated on human landings, as mosquitoes were collected at various locations covering the whole village area, and not at fixed sites such as the cave or the 200 l-drums. In that case, the HBI is 44%, larger than the overall HBI of 26.6% which represents a kind of "average proportion fed on man". Taking into account the numerous factors of variation in HBI computation, the HBI estimate for Mataral would lie between 30–50%.

If the mosquito species is not highly anthropophilic, the HBI may vary from one location to another, depending on the proportion of humans and other possible hosts living in the areas and the availability of these hosts. It is not always possible to generalize an estimated value to other areas, even close. For example, in 3 villages of Venezuela HBIs varied 0–50% for each of the anopheline species studied [33]. In Papua New Guinea HBIs for *Anopheles farauti *or *Anopheles punctulatus *ranged from 9 – 83% in villages within a 20 km radius [34,35]. For *An. pseudopunctipennis*, old (and not very accurate) data give estimates ranging from 2 – 85% depending on countries (40–80% in Peru [13,14], 50% in Argentina [10], 67% in Mexico [12] and as low as 2% in Venezuela [11]. Despite the lack of precision of these data, the underlying conclusion is that the HBI appears to be variable from one place to another. So it is better to refer to the HBI of a population than to that of a species as a whole.

In the range of distribution of *An. pseudopunctipennis *in central Andes of Bolivia, villages are similar to Mataral and one can expect to encounter similar HBI values, i.e. estimates ranging from 30–50%. Even crude, this estimation is far below HBI estimates of efficient malaria vectors such as *An. gambiae *or *An. funestus *which are commonly >90% [31]. As such, *An. pseudopunctipennis *enters the "opportunistic" category of mosquitoes as opposed to "fixed" species as regard to host preference [6], which include highly antropophilic species (such as *An. gambiae*) or zoophilic species at the other end of the spectrum (such as *An. quadrimaculatus*) [36].

In terms of host preference, accurate interpretation of mosquito blood-feeding patterns cannot be made unless data are also available on densities of the various possible hosts present in the study area. Forage ratios enable converting such data to indices of host preference. They distinguish species which have "fixed" feeding preferences from those which exhibit "opportunistic" behaviour. They point out preferences (and avoidance), and also quantify the degree of opportunistic behaviour and/or the degree to which host specific feeding patterns are obligate. A strictly anthropophilic species would only have "humans" as preferred hosts while an opportunistic species (such as *An. pseudopunctipennis*) will exhibit more than one preferred host. The number of significant "preferred" hosts is a measure of the opportunistic behaviour of the mosquito, and the value of the forage ratio quantifies the intensity of preference. The difficulty in using forage ratios is obtaining accurate population estimates of available hosts. However, even with rough population estimates, forage ratios are potentially more powerful indices than other specialized ones [37] for studying mosquito feeding preferences. Forage ratios may be used to compare situations in different areas (for example the choice of humans by *An. pesudopunctipennis *in various villages) or to compare various mosquito species.

In Mataral, experiments carried out with baited mosquito-nets show that "man" is seldom selected in first, except when only pigs, dogs and Chicken are the only other hosts offered (August 2002 experiment). *An. pseudopuntipennis *does not seem to be strongly attracted to humans. Pigs, chickens and dogs seem to be the less chosen hosts. On the other hand, sheep, goats and donkeys are the most appreciated (followed by "humans" and "cows"). So, as demonstrated by forage ratios, *An. pseudopunctipennis *make host choices at least in two categories: "preferred hosts" (goats, donkeys, humans, cows) and "avoided hosts" (dogs, chicken, pigs). If *An. pseudopunctipennis *do make host choices, the intensity of preference depends on what species of hosts are competing: If all the preferred species are absent, *An. pseudopunctipennis *will feed on whatever host is encountered.

The only experiment when "man" has been chosen as the first host choice was in May 2003 and curiously was opposed to the usually favourite "donkey" and "sheep/goat". This result points out the large variations that may exist in host choice and the likely role of seasonal environmental varying factors. Nevertheless, even in that last experiment, the proportion of mosquitoes attracted to man was only 51.4% (weighted proportion 45.1%) indicating that *An. pseudopunctipennis *is not very anthropophilic. The opportunistic behaviour of *An. pseudopunctipennis *is also illustrated by the variations of the HBI values within the sampling locations. In that case, HBIs reflect the availability of the closest preferred host and also that, amongst the "preferred" group, the opportunistic behaviour of *An. pseudopunctipennis *may be characterized as "first preferred host encountered, first bitten".

Because of the weakly anthropophilic behaviour of *An. pseudopunctipennis*, the dozens of goats and sheep raised by the villagers in the central Andes that are kept close to houses at night may act as a zooprophylaxis barrier to malaria transmission. *De facto*, malaria transmission is less active in the Mataral area than in the Yacuiba area (south of Bolivia) where less cattle is raised and where cattle do not sleep close to human habitations (pers. observations). The question is then to quantify in what proportion the ratio humans/animals has to change to significantly diminish the HBI and thus the malaria transmission.

A poor HBI is one of the cue factors that diminish the vectorial capacity of *An. pseudopunctipennis*. Moreover, the proportion of parous females attracted to humans is not higher than the proportion of nulliparous indicating that transmission risk does not increase when the mosquito moves from one physiological category to the other. Neither seems to increase the proportion of mosquitoes that came back to bite humans when they already have taken a bloodmeal on man during the preceding trophic cycle or during multiple feedings. This proportion is 44%, in the range of the estimated HBI. In that group, the proportion of parous females (i.e., that are potentially dangerous for malaria transmission) is not different in those that came back to humans than in those who bite animals. The population of *An. pseudopunctipennis *from Mataral does not seem to be separated into two groups, one anthropophilic and the other more zoophilic.

Seasonal shift from feeding on mammals to birds has been reported in some *Culex *species [38-42]. In Mataral, with time, there was a slight increase in the monthly HBI values from January to October. In the two sampling situations (cave and human landings), the rate of increase was the same (regression lines were significant and parallel). However these calculations were carried out with small numbers (30 to 70 mosquitoes for each month and for each situation), and so the conclusion may be wrong. If not, the tendency is not well understood, as there is no evident correlation between temperature, rainfall or other environmental parameter variations and the increase in the proportion of blood-feedings on humans rather than on other mammals.

Except with cows, multiple bloodmeals distribution and the high proportion of them are in accordance with the opportunistic behaviour of the mosquito. The over-representation of mixed meals taken on cows are a consequence of the combination of the host preference (high forage ratios) with the few animals present in Mataral. If mixed meals are not from immediate successive meals, their high proportion in Mataral and their high proportion in the sub-population of mosquitoes captured on human landings indicate that the duration of the gonotrophic cycle of *An. pseudopunctipennis *may have a large variance and/or that this mosquito (or at least some individuals) may often need more than one bloodmeal to complete egg maturation. Mixed meals originated from interrupted feeding are highly frequent as 51% of the mosquitoes which are in Christopher's stage II or with fresh incomplete bloodmeals when captured had already a bloodmeal from an animal when coming to feed on volunteers. All proportions calculated with patent mixed meals may be under evaluated because cryptic meals were not taken into account in these computations (no attempt has been made to estimate exactly the proportion of cryptic meals). However, data on human landings suggest that maybe >40% of bloodmeals taken on humans could be cryptic. Cryptic meals are of epidemiological interest as they come from feedings on two or more hosts of the same species. Some argue that probing may in fact diminish the probability that a mosquito becomes infected (and then infective) as the total number of gametocytes ingested will be less for a partial meal, and the probability for the same mosquito to complete its meal on another gametocytemic person is low [35,43]. However, if the mosquito is infective, probing may increase the probability for one person to become infected. As such, cryptic meals increase the classic definition of vectorial capacity [22]. The likely high proportion of cryptic meals on humans as calculated from human landings in Mataral is in accordance with other findings [35]. Research is still needed to quantify the heterogeneity of cryptic meals in one group of hosts (humans for example). Heterogeneity may have a strong impact on transmission dynamics if, for example, mosquitoes are attracted more to one (e. g. gametocytaemic) category than to another one amongst the group [44,45].

In the central Andes, villages are dispersed and the human population as well as other mammals populations remain scarce. *An. pseudopunctipennis *cannot survive in such habitats if it has a high specialized host preference (to humans for example). Moreover, *An. pseudopunctipennis *is also found in unpopulated areas indicating that, without humans, it may feed on other (wild?) mammals. The recent historical settlement of the central Andes suggests that *An. pseudopunctipennis *has been in contact with humans for only a few centuries and so still keeps its "opportunistic" (zoophilic-based?) habits. However, when humans are present the mosquitoes feed on them with an HBI of 30–50%. This range of values for *An. pseudopunctipennis *in the central Andes area is similar to that of weak malaria transmitting species in other parts of the world [16]. From the transmission viewpoint, this weakness may be compensated by other parameters such as high female densities that can enhance the vectorial capacity. *An. pseudopunctipennis *is well adapted to its varying environment: it is ubiquist in the choice of its larval habitats, is active even at low temperatures and can quickly reconstitute high adult densities. *P. vivax *is so far the only human malaria parasite circulating in the central Andes. The extrinsic cycle of the parasite is shorter than that of *Plasmodium falciparum *and the parasite presents hypnozoites stages that can remain dormant in the liver of humans for extended periods (months or years) before reactivating and invading the blood. These characteristics enable a weak vector such as *An. pseudopunctipennis *to activate a transmission cycle, even during short periods, as soon as ecological and population conditions are adequate.

The pair *An. pseudopunctipennis/P. vivax *demonstrate a good level of co-adaptation in the central Andes. Weak transmission points of the mosquito (such as a low HBI) and fluctuating environmental conditions are "compensated" by intrinsic biological factors of the parasite. Malaria in the central Andes is then unstable in the sense of Macdonald [4], with determining causes such as low vector antropophily and climatic conditions favourable for short periods of transmission. Due to difficulties in estimating all the components of the vectorial capacity (including the HBI), the risk of malaria transmission is often measured with more simple indexes for which the estimation of the HBI is not needed. For example, the entomological inoculation rate (EIR), defined as EIR = *m.a.s*, where *m *and *a *are as usual, and *s *is the sporozoite rate (proportion of mosquitoes with sporozoites) is often used. The product *m.a *may simply be estimated by human bait collections and in itself may serve as a rough index of transmission risk. In combination with the proportion *P *of parous females, the measurement of *m.a.P *(the number of parous females biting per human per night) gives also an interesting index of transmission, as well as *m.a *alone in some situations. In those cases, the direct estimation of *a *is not necessary. However, the understanding of mosquito feeding habits, host choices and the computation of the HBI (even crude), are essential to know the conditions under which a mosquito may be distracted from its preferred hosts. Some vector control techniques such as zooprophylaxy [4,24,46,47] or inside residual insecticide spraying [48] have an impact on the HBI. The degree of variation may be one of the possible parameters to investigate in the follow-up of transmission patterns. To achieve this, adequate sampling techniques should be implemented in time and space to estimate the HBI with precision and distinguish confounding factors that may affect its estimation [49]. Samples should be taken from various sites, avoiding the introduction of bias due to collections too close from specific sources of blood. The geographic distribution of engorged resting females in the field is a cue factor. Unfortunately, this distribution is not random, even after some time following the bloodmeal enabling mosquitoes to move off the blood sources and disperse in nature. The HBI should then be computed using a weighted mean taking into account all the selected sites of the sample. Weighting should take into account what proportions of the blood-fed females of the species are resting in each type of habitat, which may be difficult to assess. For that reason, the estimated HBI for *An. pseudopunctipennis *in the central Andes was estimated with a range and not as an exact figure.

## Conclusion

Forage ratios are powerful tools to interpret mosquito feeding host choices. They can also be used to detect changes in HBI over time in one place or to compare various situations. However, sampling engorged mosquitoes is not always easy as HBI calculations need representative samples of the vector population. Research is still needed to derive accurate sampling strategies adapted to HBI estimation. Mosquito host choices depend on various factors which can be grouped in two suggested categories depending on the mosquito itself (the "biting power" concept) and on its environment ("availability" concept). On the basis of the suggested divisions of these parameters (availability = accessibility + vulnerability; biting power = host preference + biting efficiency + biting effectiveness) it would be interesting to identify and quantify the confounding factors of mosquitoes host choice and design a standard sampling strategy for HBI estimation. However, our results showed that *An. pseudopunctipennis *in the central Andes is not very anthropophilic (HBI ranged from 30–50%), in accordance with the unstable malaria transmission scheme in that area. Because *An. pseudopunctipennis *may choose amongst preferred hosts, an alternative vector control strategy may use this behaviour to attract mosquitoes to insecticide and kill them before they transmit. In addition to all other control methods used, this prophylaxis approach should be considered.

## Authors' contributions

LF Designed the study, carried out the field work, taught LP to process blood-fed mosquito samples with ELISA, analysed the data and wrote the manuscript.

LP Processed the blood-fed mosquito samples (ELISA)

BB Helped in designing the study and in collecting mosquitoes in the field.

CT Helped in the field work and in processing ELISA samples.

All authors erad and approved the final manuscript.
